# Utility of wound cultures in the management of open globe injuries: a 5-year retrospective review

**DOI:** 10.1186/s12348-020-0196-5

**Published:** 2020-02-03

**Authors:** Ryan T. Drumright, Kathleen A. Regan, Albert L. Lin, Meghan G. Moroux, Siva S. R. Iyer

**Affiliations:** 1 Murfreesboro Medical Clinic, 1272 Garrison Dr, Murfreesboro, TN 37129 USA; 2 Department of Ophthalmology and Visual Sciences, University of Wisconsin School of Medicine and Public Health, 2880 University Avenue, Madison, WI 53705 USA; 3 Department of Ophthalmology, University of Mississippi School of Medicine, 2500 N State Street, Jackson, MS 39216 USA; 4 Department of Ophthalmology, Georgetown University, Washington DC. 3800 Reservoir Road, Washington, DC 20007 USA; 5 Department of Ophthalmology, University of Florida College of Medicine, 1600 SW Archer Rd, Gainesville, FL 32610 USA

**Keywords:** Globe trauma, Endophthalmitis, Culture, Open globe injury

## Abstract

**Background:**

Endophthalmitis after open globe injury can be devastating to vision recovery. As treatment of endophthalmitis is often empiric, some surgeons may obtain cultures at presentation of trauma in anticipation of later infection. This study examines the usefulness of wound cultures obtained during globe repair.

**Results:**

Institutional Review Board approval was obtained. Medical records were retrospectively reviewed, with 168 open globes included. Cultures of the wound site had been taken in all cases included in this study. Wound cultures were positive in 63% of cases but were not used for clinical decision-making for any patient in this study. Two patients had evidence of endophthalmitis at presentation, with results of vitreous culture matching those from the wound. No patient later developed endophthalmitis after open globe repair.

**Conclusions:**

Despite a high rate of wound contamination, few cases of endophthalmitis (1.2%) were seen in this series. In no case did the results of wound culture impact choice of antibiotic prophylaxis or treatment. Cultures obtained at the time of open globe repair were not cost effective in the subsequent management of the injury.

## Background

Open globe injury results from penetration or perforation in sharp or projectile trauma or from rupture in blunt trauma [1]. Endophthalmitis is a potentially devastating sequela of open globe injury. Rates of endophthalmitis after severe ocular trauma range from 0 to 17% [2, 3], with a higher risk in the presence of an intraocular foreign body (IOFB) [4, 5]. Outcomes are typically worse than post-operative endophthalmitis [2].

Prophylactic antibiotics may be administered, but data supporting a particular treatment protocol are scarce [6]. If endophthalmitis develops, treatment is often initiated empirically [7]. *Staphylococcus* and *Streptococcus* species are common causative agents [7], as they are skin flora and may enter through an open wound [2]. Some surgeons may obtain samples for culture at the time of globe repair to have a responsible microbial agent identified by the time the clinical picture worsens [8, 9]. However, despite positive cultures even from intraocular sources at the time of open globe repair, endophthalmitis may not develop [8, 10].

Some surgeons, including those at our institution, routinely obtain wound cultures at the time of surgical repair [7, 8]. The purpose of this study was to analyze the clinical usefulness and cost-effectiveness of this test in the care of open globe injury.

## Methods

Institutional review board (IRB) approval was obtained at the University of Mississippi. This study was a retrospective non-comparative case series patient record review from June 2012 to April 2016. Electronic medical records were searched for a diagnosis code of open globe or corneal-scleral laceration and reviewed for correct coding. Patients who underwent primary globe repair, had wound cultures taken preoperatively, and followed up for at least 1 month post-operatively were included. Patients were excluded if the eye was primarily removed rather than repaired, if cultures were not obtained, or if they were lost to follow-up prior to 1 month.

After induction of general anesthesia and endotracheal intubation, cultures were obtained from the wound site using cotton tip applicators. See Table 1 for listing of cultures obtained as a departmental standard in every case. The globe was then prepped with 5% ophthalmic betadine and draped for ophthalmic surgery. Globe repair was performed by multiple faculty at a single institution with a variety of approaches corresponding to the nature of the injury. Ocular and systemic antibiotics were administered at the discretion of the surgeon. If given, ocular antibiotics were administered after cultures were obtained. Patients were started on topical medication post-operatively (prednisolone acetate 1%, moxifloxacin 0.5%, atropine 1%) for at least the following week and were followed in clinic.

**Table 1:** Culture media used and cost of supplies

Culture media	Cost (direct from supplier)
Blood agar	$4.40
Chocolate agar	$4.00
MacConkey agar	$3.07
Sabouraud Dextrose agar slant	$2.81
Sabouraud Dextrose with Chloramphenicol agar slant	$2.14
Mycosel agar slant	$1.47
Lowenstein-Jensen agar slant	$7.50
Thioglycollate broth with hemin and Vitamin K	$0.88

**Fig. 1:**
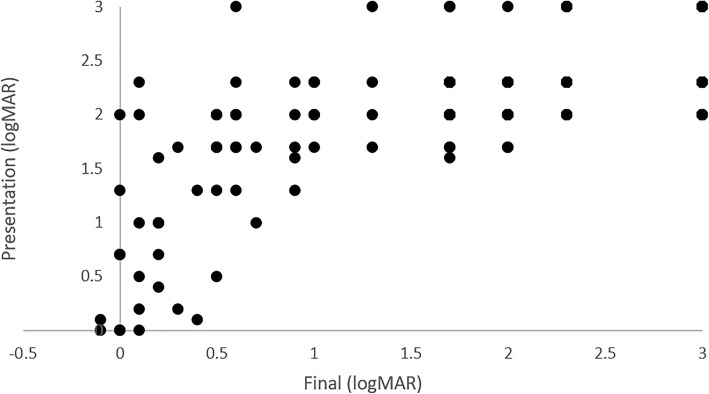
Visual acuity at presentation and at final follow-up. Vision is provided in logarithm of minimum angle of resolution (logMAR)

For statistical analysis, Snellen visual acuity was converted to logarithm of the minimum angle of resolution (logMAR) scale (Fig. 1). Nonnumeric vision was converted as follows [11]: count fingers (CF) 1.7, hand motion (HM) 2.0, light perception (LP) 2.3, and no light perception (NLP) 3.0.

## Results

### Patient presentation

Two hundred and twenty-nine eyes were recorded with a diagnosis code of open globe injury. Eleven eyes were primarily enucleated or eviscerated, 39 were lost to follow-up prior to 1 month, and 11 did not have cultures obtained. Remaining 168 eyes of 166 patients were included for study, including both eyes in two cases of bilateral ocular trauma.

The average patient age at presentation was 38 years (range 1–93). Average length of follow-up was 272 days. There were 42 females (25%). Spring (March–May) was the most common season of presentation, with 53 globes (31.5%); winter (December–February) and summer (June–August) were next most common, with 41 and 43 globes, respectively (24.4 and 25.6%); and fall (September–November) least common with 31 globes (18.5%). An intraocular foreign body was found in 23 eyes (13.7%). Biomaterial was involved in 50 globes (29.8%). Most common mechanisms of injury were metal (26.8%) and wood or plant material (13.1%). Assault and fall were also common causes, each responsible for 11.3% of cases (Table 2).

**Table 2:** Mechanism of open globe trauma

Mechanism	# of eyes	% of total
Metal	45	26.8
Wood/plant	22	13.1
Assault	19	11.3
Fall	19	11.3
Bullet/BB	17	10.1
Glass	10	6.0
Motor vehicle collision	10	6.0
Finger	7	4.2
Other	13	7.7
Unknown	6	3.6

### Antibiotic choice

Antibiotics were administered in a majority of cases (Table 3). Intraocular antibiotics were administered in 151 eyes (90%). Vancomycin was administered in each of these cases, usually in combination with clindamycin (79%) but with ceftazidime in 11% and by itself in one case. Subconjunctival antibiotics were administered in 88% of cases. Tobramycin was the most common subconjunctival choice; cefazolin, vancomycin, and ceftazidime were also administered. Systemic antibiotics were administered in the emergency department or operating room in 79% of cases. Cefazolin was the most common (76%); and ceftazidime, ceftriaxone, and vancomycin were less frequently administered.

**Table 3:** Ocular and systemic antibiotics administered at time of ruptured globe repair

Antibiotic	# of eyes	% of total
Intraocular	151	89.9
Vancomycin	151	89.9
+ Clindamycin	132	78.6
+ Ceftazidime	18	10.7
Subconjunctival	147	87.5
Tobramycin	145	86.3
Cefazolin	10	6.0
Vancomycin	3	1.8
Ceftazidime	2	1.2
Systemic	132	78.6
Cefazolin	127	75.6
Ceftriaxone	3	1.8
Ceftazidime	2	1.2
Vancomycin	1	0.6

### Culture results

Overall, 106 (63%) of cultures obtained were positive. Of these positive cultures, *Staphylococcus* species were most commonly seen (91.5%), *Streptococcus viridans* was next most common (10.4%), and 17.9% were polymicrobial. Only five samples grew fungus (Table 4). Mechanism of injury involving biomaterial was not significantly associated with culture positivity of trauma without biomaterial (*p* = 0.15 by Chi square).

**Table 4:** Infectious agents in open globe trauma

Organism	# of Samples	% of positive cultures
Pathogenic		
*Staphylococcus* spp.	97	91.5
*Coagulase-negative Staphyloccocus*	96	
MSSA	4	
MRSA	1	
*Streptococcus viridans*	11	10.4
*Bacillus* spp.	6	5.7
*Cladosporium*	3	2.8
*Diphtheroids*	2	1.9
*Micrococcus luteus*	2	
*Moraxella catarrhalis*	2	
*Enterococcus faecalis*	2	
*Pseudomonas* spp.	2	
*Klebsiella pneumoniae*	1	0.9
*Morganella morganii*	1	
*Haemophilus influenzae*	1	
*Cryptococcus laurentii*	1	
*Enterobacter cloacae complex*	1	
*Acinetobacter baumannii*	1	
Non-pathogenic		
*Syncephalastrum racemosum*	1	
*Leclercia adecarboxylata*	1	
Summary		
Polymicrobial	19	17.9

### Development of endophthalmitis

Two patients presented with evidence of endophthalmitis at the time of their open globe injury (1.2%). Neither had an IOFB. In addition to wound cultures, vitreous samples were obtained during their open globe repair either by pars plana vitrectomy or by vitreous tap. Both patients grew coagulase-negative staphylococcus on both their wound and intraocular cultures. None of the remaining 227 eyes screened for this study developed endophthalmitis, and their culture results were not used for the remainder of their care. In no case was topical antibiotic prophylaxis changed based on culture results.

Wound culture, in this study, had a 100% sensitivity, a 37.4% specificity, and a positive predictive value of 1.9% for endophthalmitis development, with an accuracy of 38%.

### Culture costs

Cost of culture media from the supplier were calculated for the standard culture analysis performed per patient (Table 1).

Per patient, total cost for supplies was $26.27, and hospital charges for cultures were $875. This amounts to $2206.68 of supplies and $73,500 of patient charges per case of endophthalmitis secondary to an open globe.

### Vision outcomes

Of the 168 eyes, 17 did not have a recordable vision secondary to patient cooperation with exam. Of those with recordable vision, mean vision was HM (logMAR 2.1) at presentation and CF (logMAR 1.8) at last follow-up. At final follow-up, vision improvement was seen in 66 eyes (43.7% of recorded), 40 worsened (26.4%), and 45 were stable (29.8%) compared with presentation. The eye was eviscerated or enucleated in 21 cases (12.5%) during the follow-up period. Final vision was correlated with vision at presentation (*r* = 0.75, *p* < 0.05).

## Discussion

Swab cultures from the globe wound were obtained preoperatively in all cases included in this study. The culture results obtained were not used clinically, and no patient without evidence of infection at presentation went on to develop endophthalmitis. For the two patients with endophthalmitis at presentation, vitreous cultures were obtained that matched wound cultures.

To the best of our knowledge, our study represents the largest study to date of wound cultures in open globe injuries. Wound cultures were positive in 63%, compared with 23% by Rubsamen et al. [8] and 20% of conjunctival wash cultures by Bhala et al. [12]. Rubsamen et al. found a low sensitivity but high specificity of wound cultures obtained intraoperatively [8]. In that study, the rate of traumatic endophthalmitis was 13% and the intraoperative cultures were clinically useful for antibiotic selection [8]. The study by Bhala et al. [12] also reported a high rate of post-traumatic endophthalmitis (40%), and a positive culture obtained at the time of globe repair correlated to risk of infection. No distinction was made, however, between positive intra- or extra-ocular culture result in endophthalmitis risk [12]. Conjunctival and eyelid swab cultures may correlate with aqueous cultures at the time of globe repair, indicating that contamination may be from the skin flora prior to or during surgery [9].

Open globe repair has been reported to cost $850–3000 [13–15] in hospital charges worldwide, with higher costs for more complex cases requiring further surgery [14]. Post-operative hospitalization further increases cost, up to an average of $4500 [14]. The societal impact can be devastating, as vision or globe loss can lead to disability and an estimated loss of 25% of earning capacity [16]. Globe loss during war, for example, is estimated to cost $3 million over a lifetime [17].

Endophthalmitis, too, can be very costly to the healthcare system. Post-operative endophthalmitis increases Medicare charges by $3500 [18]. Prophylactic intraocular antibiotics, however, may save the healthcare system $88,000 over 10 years [19].

Intraocular antibiotics has been shown by meta-analysis to reduce the risk of traumatic endophthalmitis [20]. While several centers routinely use prophylactic systemic antibiotics [5, 21, 22], their use is controversial without strong supporting evidence [7]. Intravenous antibiotics were not advantageous over oral prophylaxis in a randomized controlled trial [6]; however, systemic antibiotics have not been compared with local administration in a similar fashion. Systemic antibiotics may have a poor intraocular penetration, and intravenous therapy increases hospitalization costs [23].

Patients in this study were not randomized, and the majority of patient received ocular and systemic antibiotics per surgeon preference. Although a prospective study would not utilize the insurance system for culture costs, this retrospective study collected data from an established treatment approach in place as a departmental standard. The rate of endophthalmitis seen in this study is on the lower end of the range reported in the literature [2], possibly related to the high use of prophylactic antibiotics.

## Conclusions

In conclusion, cultures obtained at the time of open globe injury repair during this study period were not clinically useful or cost effective in the subsequent management of the injury. With a low rate of endophthalmitis, the positive predictive value of this test was low. We recommend obtaining cultures only if evidence of intraocular infection exists.

## Data Availability

The datasets used and/or analyzed during the current study are available from the corresponding author on reasonable request.

## Abbreviations

CF: Count fingers
HM: Hand motion
IOFB: Intraocular foreign body
IRB: Institutional review board
logMAR: Logarithm of the minimum angle of resolution
LP: Light perception
NLP: No light perception
